# Discovery of novel non-peptidic and non-covalent small-molecule 3CL^pro^ inhibitors as potential candidate for COVID-19 treatment

**DOI:** 10.1038/s41392-023-01482-9

**Published:** 2023-05-22

**Authors:** Zhidong Jiang, Bo Feng, Yumin Zhang, Tianqing Nie, Hong Liu, Jia Li, Haixia Su, Leike Zhang, Yi Zang, Yu Zhou

**Affiliations:** 1 School of Life Science and Technology, ShanghaiTech University, Shanghai, 201203 China; 2 Lingang Laboratory, Shanghai, 201203 China; 3 Shanghai Institute of Materia Medica, Chinese Academy of Sciences, Shanghai, 201203 China; 4 Shenyang Pharmaceutical University, Shenyang, 110016 China; 5 State Key Laboratory of Virology, Wuhan Institute of Virology, Center for Biosafety Mega-Science, Chinese Academy of Sciences, Wuhan, 430071 China

**Keywords:** Medicinal chemistry, Drug development

**Dear Editor**,

The global coronavirus disease 2019 (COVID-19) pandemic, which is caused by severe acute respiratory syndrome coronavirus 2 (SARS-CoV-2) has brought a profound impact on humanity. SARS-CoV-2 enters host cells through the interaction of its spike glycoprotein with angiotensin-converting enzyme 2 (ACE2) of the host and releases its viral RNA genome.^1^ The genome is further translated into two polyproteins, PP1a and PP1ab, that contain 16 non-structural proteins, in which the non-structural protein 5 (also called as 3C-Like protease, 3CL^pro^) plays a vital role in the life cycle of the virus.^2^ As a consequence, multiple peptide-like covalent 3CL^pro^ inhibitors have been successively reported to show good 3CL^pro^ inhibitory potencies and antiviral activities.^3,4^ However, the defects of peptidomimetic inhibitors about low membrane permeability and metabolic stability are obvious, in which the approved drug paxlovid (PF-07321332) from Pfizer must be co-administrated with ritonavir as a pharmacokinetic booster.^3^ Non-peptidic small molecule inhibitors may have more advantages in metabolic properties and are gradually attracting attention.^5–7^ Our research attempted to discover a novel non-peptidic and non-covalent small molecule 3CL^pro^ inhibitor so as to give better drug-like properties and good antiviral activities.

After screening more than 1,700,000 compounds from the Chinese National Compound Library,^8^ we identified 1851 hits targeting 3CL^pro^. Taken together with molecular docking and cell-based antiviral assay, we selected two structurally similar compounds, **G006** and **G004**, for further optimization (Fig. 1a). The docking analyses for **G006** demonstrated that the quinoline-2-one occupies the S1 site to form an important hydrogen bond with Glu166 and Gly143, the 3-chlorophenyl occupies the hydrophobic S2 site to form a π–π interaction with His41. (Fig. 1b and Supplementary Fig. 1). As we have seen, the active site was not fully occupied by **G006**, which may limit its inhibition potency against 3CL^pro^ (Fig. 1b). Thus, we have attempted to introduce diversified side chains onto the core piperazine ring in the hope of filling these unoccupied cavities fully. Finally, **JZD-07** outstripped its analogs, exhibiting excellent inhibitory activity against the 3CL^pro^ of both SARS-CoV-2 and SARS-CoV-1, with IC_50_ of 0.15 μM and 0.11 μM, respectively (Fig. 1c).

**Fig. 1:**
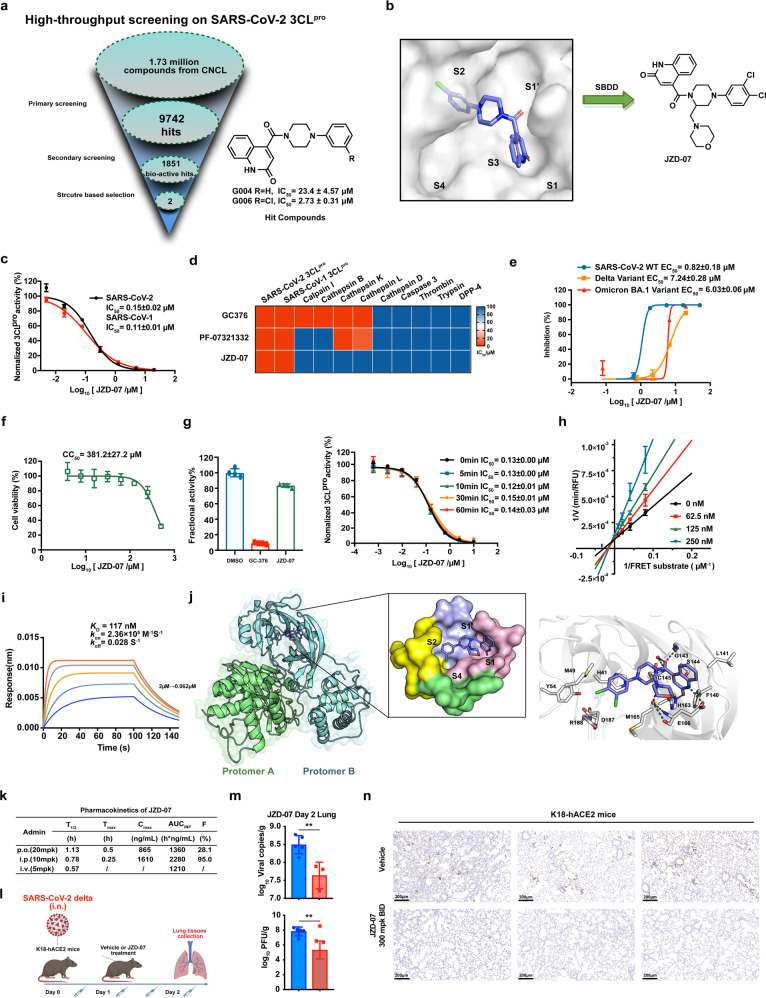
Discovery of Novel Non-peptidic and Non-covalent Small Molecule SARS-CoV‑2 3CL Protease Inhibitors. **a** Flowchart of the High-throughput screening and hit validation process. **b** Docking of lead compounds with SARS-CoV-2 3CL^pro^ and rationale design for the noncovalent SARS-CoV-2 3CL^pro^ inhibitor. **c** Compound **JZD-07** is a potent inhibitor of SARS-CoV-2 3CL^pro^ as well as SARS-CoV-1 3CL.^pro^ **d** Biochemical assay of several human proteases, compound **JZD-07** showing great selectivity against human protease, especially Calpain I and Cathepsin family protein, the values plotted were the IC_50_ values from the FRET-based enzymatic assay shown as a heatmap. **e** Inhibition of original strain (blue), Delta variant (yellow), and Omicron BA.1. variant (red) replication by Compound **JZD-07** in Vero E6 cells. Data are shown as mean ± SD, *n* = 3 biological replicates. **f** Cytotoxicity (CC_50_) to Vero E6 cells measured by CCK-8 assays. Data are shown as mean ± SD, *n* = 3 biological replicates. **g** Rapid dilution assay and time-dependent inhibition of SARS-CoV-2 3CL^pro^ by Compound **JZD-07**. A 40 nM SARS-CoV-2 3CL^pro^ was preincubated with Compound **JZD-07** for various periods of time (0 min to 1 h) before the addition of 20 μM FRET substrate to initiate the enzymatic reaction. Data are shown as mean ± SD. **h** Lineweaver-Burk plot depicting the mode of inhibition for Compound **JZD-07** on SARS-CoV-2 3CL^pro^ activity using different inhibitor concentrations. **i** Compound **JZD-07** to SARS-CoV-2 3CL^pro^ measured by biolayer interferometry (BLI), BLI analysis showing the representative association and disassociation curves of Compound **JZD-07** at series concentrations. **j** Binding mode of **JZD-07** (PDB ID: 8GTV) with SARS-CoV-2 3CL^pro^ revealed by co-crystal structure. The two protomers are shown in the green (protomer A) and cyan (protomer B) cartoon, respectively. **JZD-07** is shown as slate sticks. The surface representation of **JZD-07** interacting with S1ʹ-S4 subsites of SARS-CoV-2 3CL^pro^. The subsites are colored light blue (S1ʹ), light pink (S1), yellow (S2), and green (S4). Interactions of **JZD-07** with the surrounding residues are also revealed by the crystal structures. Residues (gray), as well as **JZD-07**, are shown in the sticks. H-bonds are represented by black dashed lines. **k** Preliminary PK evaluation of compound **JZD-07** in mice. Dosed orally at 20 mg/kg. Dosed intraperitoneally at 10 mg/kg. Dosed intravenously at 5 mg/kg. **l** In vivo efficacy of compound **JZD-07** in K18-hACE2 mice infected with SARS-CoV-2 delta variant. Experiment flow diagram. **m** Viral RNA copies and viral titers in lung tissues of each group were determined on day 2 after the SARS-CoV-2 delta variant challenge (**P ≤ 0.01). **n** Immunohistochemistry staining targets viral nucleocapsid protein. (SARS-CoV-2 Nucleocapsid Protein (HL344) Rabbit mAb #26369, CST). Viral nucleocapsid proteins were stained in brown

To explore the selectivity of this 3CL^pro^ inhibitor, we first examined its inhibitory activities against multiple closely related proteases, which indicated **JZD-07** had almost no inhibition for human calpain I, cathepsin (B, D, K, and L), thrombin A, trypsin, caspase-3, DPP-4, and proteasome, while the control 3CL^pro^ inhibitors **GC-376** and **PF-07321332** showed certain inhibitory potencies against different cathepsin proteins (Fig. 1d). We also evaluated the inhibitory potencies toward SARS-CoV-2 viral life cycle related targets, and **JZD-07** had no inhibition against those enzymes with IC_50_ > 100 μM (Supplementary Table 1). These data demonstrate that **JZD-07** is a highly specific 3CL^pro^ inhibitor.

Based on the inhibitory potency against 3CL^pro^, we conducted an antivirus assay of **JZD-07** in Vero E6 cells. **JZD-07** showed good inhibitory activity against the SARS-CoV-2 original virus with EC_50_ of 0.82 μM and also showed dose-dependent inhibition on the replication of delta and omicron BA.1. variant with EC_50_ values of 7.24 μM and 6.03 μM, respectively (Fig. 1e and Supplementary Fig. 2). Besides, **JZD-07** exhibited good antivirus activities against HCoV-OC43 and HCoV-229E (Supplementary Fig. 2). Meanwhile, this compound almost has no effect on cell viability with the CC_50_ in Vero E6 cell over 300 μM (Fig. 1f). These results indicate that **JZD-07** has good antiviral activity in vitro and almost has no cytotoxicity.

To elucidate the mechanism of action of this non-peptidic small molecule inhibitor, the enzyme kinetics and drug binding-kinetics profiles were studied in detail. Firstly, the inhibition reversibility was investigated by rapid dilution assay. **JZD-07** (10 μM) or an irreversible 3CL^pro^ inhibitor (**GC-376**, 2 μM) were incubated with SARS-CoV-2 3CL^pro^ (2 μM) for 20 min, the activities of 3CL^pro^ incubated with **JZD-07** recovered more than 70% after 100-fold dilution. Meanwhile, the IC_50_ values did not change with preincubation time (Fig. 1g), which indicates that the inhibition against 3CL^pro^ is reversible. Furthermore, we determined the kinetic parameters using different concentrations of **JZD-07**. After global fits of the respective enzyme inhibition curves, **JZD-07** is shown to be a competitive inhibitor with *a K*_i_ value of 0.117 μM (Fig. 1h), comparable to the reported 3CL^pro^ inhibitor **CCF981** and more potent than **ML-188** (Supplementary Fig. 3). Recently, the importance of drug-binding kinetics profiles for drug design have been increasingly recognized. We utilized bio-layer interferometry (BLI) assay to measure the binding kinetics and affinity parameters for our compound (Fig. 1i and Supplementary Fig. 4). An overall *k*_on_ value of **JZD-07** is 2.36 × 10^5^ M^−1^ S,^−1^ the dissociation behavior is very slow with *k*_off_ = 0.028 S^−1^, and the *K*_D_ value of **JZD-07** is 117 nM. Taken together, the results of enzymatic inhibition and BLI data indicate that **JZD-07** is a high affinity, slow-dissociation, and reversible competitive 3CL^pro^ inhibitor.

To elucidate the precise binding modes of the inhibitor with protease, we successfully determined the crystal structure of SARS-CoV-2 3CL^pro^ in complex with **JZD-07** at a resolution of 1.80 Å (Supplementary Table 2). The binding modes showed **JZD-07** non-covalently bounded into the substrate-binding site and occupied S1’, S1, and S2 subsites (Fig. 1j). The 2-quinolinone group fitted into the S1 subsite by forming hydrogen bonds with the side chains of His163 and Glu166. The carbonyl oxygen atom between the piperazine ring and quinoline ring fixed the conformation of the flexible oxyanion loop by making hydrogen bonds with the main chain of Gly143 as well as a water molecule which simultaneously contacted with the main chain of Cys145. The *O*-dichlorobenzene ring is deeply inserted into the S2 subsite by making extensive hydrophobic interactions with multiple residues His41/Met49/Tyr54/Met165/Asp187/Arg188. Notably, it also formed π–π stacking interactions with the side chain of catalytic His41. The morpholinyl group of **JZD-07** oriented to the S1 subsite, producing water-mediated hydrogen bonds and hydrophobic interactions with the residue Glu166. These results together provided the molecular details **JZD-07** recognized by SARS-CoV-2 3CL^pro^, which is consistent with its excellent 3CL^pro^ inhibitory activities.

Considering our compound is a novel non-covalent and non-peptidic 3CL^pro^ inhibitor, we further explored the potential advantages of PK profiles. Oral administration of 20 mg/kg of **JZD-07** could give favorable PK parameters with an oral bioavailability of 28.1%. Intraperitoneal administration of 10 mg/kg of **JZD-07** also showed an excellent PK profile with an AUC of 2280 h*ng/mL and a C_max_ of 1610 ng/mL (Fig. 1k). Besides, we have launched an acute toxicity experiment, and the results indicate that **JZD-07** has a good safety profile (Supplementary Fig. 6). These favorable PK and safety characteristics indicated **JZD-07** might be suitable for the treatment of SARS-CoV-2 in vivo infection model.

In the end, we investigated the antiviral potency for **JZD-07** in K18-hACE2 mice challenged with the SARS-CoV-2 delta variant. After 2 h of challenge, the mice were injected intraperitoneally with **JZD-07** or vehicle (day 0). Then mice were administrated twice at 8 h intervals on day 1. On day 2, the mice were euthanized 4 h after receiving one compound or vehicle treatment (Fig. 1l). Lung tissues were collected for the detection of viral RNA and viral titer, histopathological analysis and immunostaining of lungs were also evaluated. Compared with the vehicle group, the viral copy loads of the lung in the **JZD-07** (300 mg/kg bid) treated group were significantly reduced by nearly 1.0 log on day 2. More importantly, the viral titers of the lung of the **JZD-07** treated group showed 2.0 log reduction (Fig. 1m). Immunohistochemistry staining targeting viral nucleocapsid protein demonstrated that **JZD-07** suppressed viral titer in mice lungs at day 2 (Fig. 1n). All these results indicate **JZD-07** has a good therapeutic potential against SARS-CoV-2 delta variant in vivo.

Therefore, we here reported our discovery of a novel non-peptidic and non-covalent small molecule SARS-CoV-2 3CL^pro^ inhibitor. Based on the hit compounds from high-throughput screening studies and a structure-based drug design strategy, compound **JZD-07** stands out and shows an excellent inhibitory potency against 3CL^pro^. It also exhibits good anti-SARS-CoV-2 activity in Vero E6 cells without cytotoxicity. Mechanistic studies and X-ray crystal structure analysis have shown that **JZD-07** is a highly selective, slow-dissociation, non-covalent, and reversible competitive 3CL^pro^ inhibitor. **JZD-07** also displays favorable pharmacokinetic profiles in mice with good plasma exposure and bioavailability. More importantly, **JZD-07** exhibits a good therapeutic potential against the SARS-CoV-2 delta variant in vivo assay. Overall, these new findings offer a good drug candidate for the clinical treatment of COVID-19.

## Supplementary information

Supplementary Materials

## Data Availability

All data supporting the findings of this study are available within the article or from the corresponding author upon reasonable request.
