# Correction: Discovery of novel non-peptidic and non-covalent small-molecule 3CL^pro^ inhibitors as potential candidate for COVID-19 treatment

**DOI:** 10.1038/s41392-023-01665-4

**Published:** 2023-10-13

**Authors:** Zhidong Jiang, Bo Feng, Yumin Zhang, Tianqing Nie, Hong Liu, Jia Li, Haixia Su, Leike Zhang, Yi Zang, Yu Zhou

**Affiliations:** 1 School of Life Science and Technology, ShanghaiTech University, Shanghai, 201203 China; 2 Lingang Laboratory, Shanghai, 201203 China; 3 Shanghai Institute of Materia Medica, Chinese Academy of Sciences, Shanghai, 201203 China; 4 Shenyang Pharmaceutical University, Shenyang, 110016 China; 5 State Key Laboratory of Virology, Wuhan Institute of Virology, Center for Biosafety Mega-Science, Chinese Academy of Sciences, Wuhan, 430071 China

**Keywords:** Medicinal chemistry, Drug development

Correction to: *Signal Transduction and Targeted Therapy* 10.1038/s41392-023-01482-9, published online 22 May 2023

In our recent review of the article [1], the author noticed that the dots (red squares) in Fig 1m were accidentally removed from its original location during figure formatting, and the related data analysis is correct. Furthermore, we checked this paper again and this situation only occurred in Fig 1m. In addition, in Fig 1c, a legend for SARS-CoV-1 is missing and we have supplemented it. For the text section, no modifications are required. The correct data are provided as follows. The key findings of the article are not affected by these corrections. The original article has been corrected. We regret any inconvenience this has caused.

1. In Fig 1c, a legend for SARS-CoV-1 is missing. The correct image is as below.
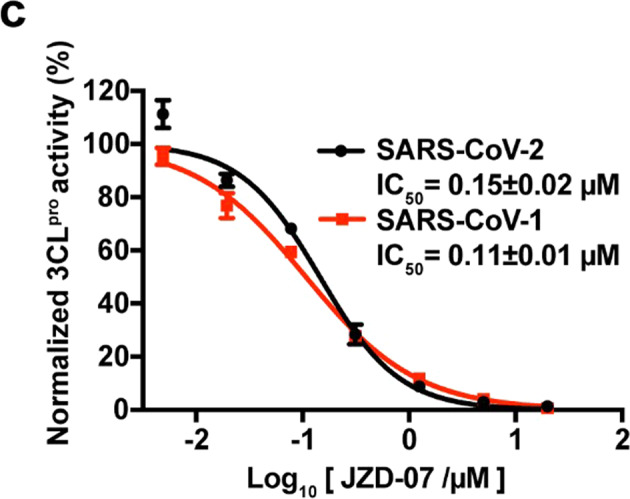
  Fig 1c Compound JZD-07 is a potent inhibitor of SARS-CoV-2 3CLpro as well as SARS-CoV-1 3CLpro.
2. In Fig 1m, the dots (red squares) were accidentally removed from its original location during figure formatting, and the related data analysis is correct. The correct image is as below.

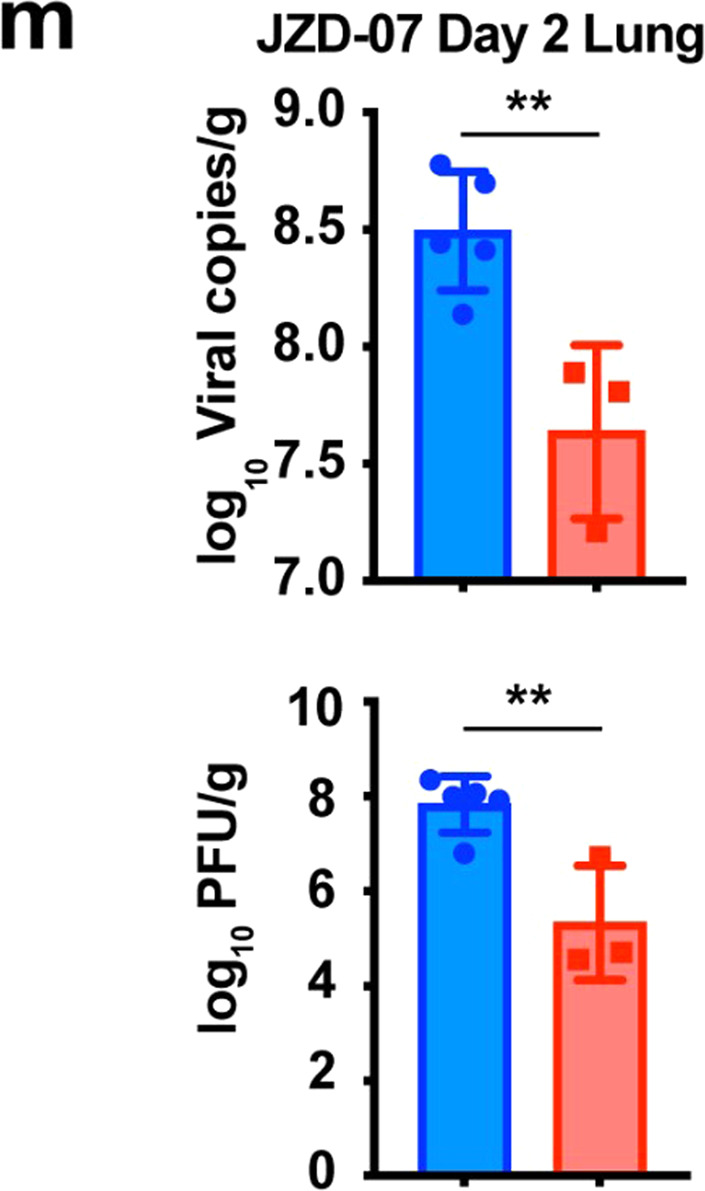


Fig 1m Viral RNA copies and viral titers in lung tissues of each group were determined on day 2 after the SARS-CoV-2 delta variant challenge (**P ≤ 0.01).

The original article has been corrected.

