# Supplementary material for: Discovery of novel non-peptidic and non-covalent small-molecule 3CLpro inhibitors as potential candidate for COVID-19 treatment
Source: Signal Transduct Target Ther. 2023 May 22;8:209. doi: 10.1038/s41392-023-01482-9 (PMC10201506; doi:10.1038/s41392-023-01482-9)
Supplement: Supplementary file 1 — Supplementary Materials [file 41392_2023_1482_MOESM1_ESM.docx]

**Supplementary Materials for**

**Discovery of novel non-peptidic and non-covalent small-molecule 3CL^pro^ inhibitors as potential candidate for COVID-19 treatment**

Zhidong Jiang^1,2,3^†, Bo Feng^3,4^†, Yumin Zhang^5^†, Tianqing Nie^3^†, Hong Liu^3^, Jia Li^3,4^, Haixia Su^3*^, Leike Zhang^5*^, Yi Zang^1,2,3*^ and Yu Zhou^1,2,3*^

^1^School of Life Science and Technology, ShanghaiTech University, Shanghai, China;

^2^Lingang Laboratory, Shanghai 201203, China;

^3^Shanghai Institute of Materia Medica, Chinese Academy of Sciences, Shanghai 201203, China;

^4^Shenyang Pharmaceutical University, Shenyang 110313, China;

^5^State Key Laboratory of Virology, Wuhan Institute of Virology, Center for Biosafety Mega-Science, Chinese Academy of Sciences, Wuhan 430071, China.

†These authors have equal contribution to this study

^*^ Corresponding author: Haixia Su (suhaixia1@simm.ac.cn) or Leike Zhang (zhangleike@wh.iov.cn) or Yi Zang (yzang@lglab.ac.cn) or Yu Zhou (zhouyu@simm.ac.cn)

**This word file includes:**

Materials and Methods

Supplementary Figs. 1 to 6

Supplementary Tables 1 to 2

Materials and Methods

African green monkey kidney Vero E6 cells (ATCC-1586) were maintained in Dulbecco’s modified Eagle’s medium (DMEM) with 10% fetal bovine serum (FBS) and 1% penicillin–streptomycin antibiotics. Cells were kept at 37 °C in a 5% CO_2_ atmosphere. The original strain 2019-nCoV-WIV04 (IVCAS6.7512), Delta variant (IVCAS6.7585), and Omicron variant B.1.1.529 (IVCAS 6.7600) of SARS-CoV-2 were obtained from National Virus Resource Center, and were propagated in Vero E6 cells.

PF-07321332, ML-188, CCF981, GRL0617 and Suramin sodium salt were purchased from MedChemExpress. GC-376 was purchased from CSNpharm. For in vitro assays, compounds were dissolved in analytical grade dimethyl sulfoxide (DMSO) to 10 mM stock solution.

SARS-CoV-1/2 3CL^pro^ Inhibition assay

The enzyme activity of SARS-CoV-1/2 3CL^pro^ was measured using FRET substrate, the recombinant SARS-CoV-1 3CL^pro^ and SARS-CoV-2 3CL^pro^ (at a final concentration of 80nM and 40nM, respectively), was mixed with each compound in 50 μL assay buffer (20 mM Tris, pH 7.3, 150 mM NaCl, 1mM EDTA, 3mM DTT, 1% Glycerol, 0.01% Tween-20, 0.1% BSA) and incubated for 10 min. The reaction was initiated by adding the FRET substrate MCA-AVLQSGFRK (DNP) K (GL Biochem, Shanghai), with a final concentration of 20 μM. After that, the fluorescence signal at 320 nm (excitation)/405 nm (emission) was immediately measured by continuous 10 points for 5 min with an EnVision multimode plate reader (Perkin Elmer, USA). The initial velocity was measured when the protease reaction was proceeding in a linear fashion. The inhibitor dose−response curves were analyzed using normalized IC_50_ regression curve fitting with control based normalization.

SARS-CoV-2 PL^pro^ inhibition assay

The activity of SARS-CoV-2 PL^pro^ was also measured by a continuous 10 points fluorometric assay for 5 min. Briefly, the recombinant SARS-CoV-2 PL^pro^ (40 nM at a final concentration) was mixed with each compound in 50 μL assay buffer (20 mM Tris pH8.0, 0.01% Tween20, 0.5 mM DTT) and incubated for 10 min. The reaction was initiated by adding the substrate Z-RLRGG-AMC (GL Biochem, Shanghai) with a final concentration of 50 μM, using wavelengths of 355 nm and 460 nm for excitation and emission, measured by an EnVision multimode plate reader (Perkin Elmer, USA).

SARS-CoV-2 RdRp inhibition assay

The detection of RNA synthesis by SARS-CoV-2 RdRp complex were established based on a real-time assay with the QuantiFluor® dsDNA Dye (Promega), which contains a fluorescent DNA-binding dye that enables sensitive quantitation of small amounts of double-stranded DNA (dsDNA) in solution. The fluorescence was measured using wavelengths of 504 nm and 531 nm for excitation and emission, measured by an EnVision multimode plate reader (Perkin Elmer, USA).The assay records the synthesis of dsRNA in a reaction using a poly-U template–primer RNA with the sequences of 5’-biotin-UUUUUUUUUUUUUUUUUUUUUUUUUUUUUUAACAGGUUCUAGAACCUGUU -3’ as a template (Sangon Biotech,Shanghai China) and ATP as the nucleotide substrate. Reactions were performed in individual wells of white 384-well low volume round bottom plates. The reaction contained 50 mM Tris-HCl, pH 7.5, 50 mM Ammonium acetate, 0.5 mM MnCl2, 20 μM ATP, 0.2 μM poly-U template–primer RNA, 0.01% Tween-20.

Human protease inhibition assay

Calpain I enzymatic assay was carried out as follows: 40μL Calpain I protein (abcam catalog # ab91019) was added to 40μL of Calpain I (at a final concentration of 100nM) in reaction buffer (50 mM HEPES pH 7.5, 50 mM NaCl, 10 mM DTT and 5 mM CaCl_2_] the enzymatic reaction was iniated by adding 10 μL peptide substrate N-Suc-Leu-Leu-Val-Tyr-7-AMC substrate (Sigma-Aldrich catalog #. S6510).. The reaction was monitored with EnVision multimode plate reader (Perkin Elmer, USA) with filters for excitation at 360 nm and emission at 460 nm at room temperature for 10 min. The IC_50_ values were calculated as described in the previous section.

Cathepsin B, Cathepsin D, Cathepsin K and Cathepsin L enzymatic assay was carried out as follows: Cathepsin B, Cathepsin K and cathepsin L buffer was 20 mM sodium acetate pH 5.5 with 4 mM EDTA and 8 mM DTT. Cathepsin D buffer was 20 mM sodium acetate pH 3.5 with 4 mM EDTA and 8 mM DTT,The activity of Cathepsin B was monitored using the substrate CBZ-Arg-Arg-AMC (GL Biochem, Shanghai, China) with a final concertration of 20μM, Cathepsin D was monitored using the substrate MCA-PLGL-Dap(Dnp)-AR-NH2 (GL Biochem, Shanghai) with a final concertration of 15μM, Cathepsin K and Cathepsin L was monitored using the substrate Z-Phe-Arg-AMC (GL Biochem, Shanghai, China) with a final concertration of 20μM. the proteolysis reactions were performed at room temperature in a 384-well black polystyrene plate (Perkin Elmer, catalog no. 3575) using a final reaction volume of 50 μL. The reaction of Cathepsin B, Cathepsin K and Cathepsin L was monitored with EnVision multimode plate reader (Perkin Elmer, USA) with filters for excitation at 360nm and emission at 460 nm at room temperature for 10 min. The reaction of Cathepsin D was monitored with EnVision multimode plate reader (Perkin Elmer, USA) with filters for excitation at 320nm and emission at 405 nm at room temperature for 10 min. The IC_50_ values were calculated as described in the previous section.

Trypsin enzymatic assay was carried out in a 50 μL volume containing 100 nM trypsin in 50 mM HEPES (pH7.6), 50mM NaCl,2mM DTT reaction buffer, 50 μM CBZ-Arg-Arg-AMC (GL Biochem, Shanghai, China), The reaction was monitored with EnVision multimode plate reader (Perkin Elmer, USA) with filters for excitation at 355nm and emission at 460 nm at room temperature for 10 min. The IC50 values were calculated as described in the previous section.

Thrombin enzymatic assay was carried out as follows: Thrombin buffer was 50 mM Tris-HCl pH 8.0,150 mM NaCl 2.5 mM CaCl_2_, the activity of Thrombin was monitored using the substrate Benzoyl-FVR-AMC (Calbiochem, Darmstadt, Germany) with a final concertration of 20μM. The reaction of Thrombin was monitored with EnVision multimode plate reader (Perkin Elmer, USA) with filters for excitation at 360 nm and emission at 460 nm at room temperature for 10 min. The IC_50_ values were calculated as described in the previous section.

Proteasome activity assay was determined by previously published method^1^, Briefly, 1 μL of compound was added to 10 μL of purified human proteasome (25 μg/mL), for 10 min, and was then added to 39 μL of synthesized substrate SucLeu-Leu-Val-Tyr-AMC (50 μM) (GL Biochem Ltd., Shanghai, P.R.China) as a reference reported. The AMC of the probe was detected by monitoring the increase in fluorescence with EnVision, at a 355 nm excitation and a 460 nm emission wavelength.

The DPP-4 activity was determined by previously published method^2^, Briefly, measuring the rate of hydrolysis of a substrate Gly-Pro-AMC (Sigma-Aldrich, St. Louis, MO), and the hydrolyzed fluorescent product amidomethylcoumarin (AMC) was continuously monitored using an excitation wavelength of 360 nm and an emission wavelength of 460 nm every 60 s for 10 min using an EnVision multimode plate reader (Perkin Elmer, USA), A typical reaction contained 15 ng/mL enzyme, 150 μM GlyPro-AMC, different concentrations of the test compounds, and assay buffer (25 mM HEPES, pH 7.5, 150 mM NaCl, 0.12 mg/mL BSA) in a total reaction volume of 100 μL.

Caspase-3 activity assay was determined by previously published method^3^, Briefly, The activity assay of caspase-3 was carried out in a system of 100 μL containing 50 mM HEPES, pH 7.4, 150 mM NaCl, 1 mM EDTA, 100 M Ac-DEVD-pNA, 20 nM caspase-3, and 2 mM DTT. The rate of hydrolysis product, pNA, was monitored continuously by change of absorbance at 405 nm for 5 min, and the initial rate of hydrolysis was determined using the early linear region of the enzymatic reaction curve.

Bio-layer interferometry analysis

BLI analyses were performed at 27 °C using a ForteBio Octet Red96 bio- sensor system (ForteBio) with Super Streptavidin Dip and Read Biosensors (ForteBio,18-5019). The SARS-CoV-2 3CL^pro^ protein was mixed with biotin (Biotinylation Kit Genemore LOT#1828M) at a molar ratio of three biotins to one SARS-CoV-2 3CLpro incubated at room temperature for 30 min, and then excess biotin was removed by using a desalting column. Biotinylated SARS-CoV-2 3CL^pro^ (120 μg/mL) was immobilized onto the Super Streptavidin biosensor (ForteBio, 18-5057) for 4 min. The tips were washed with the buffer (20 mM Tris, pH 7.3, 150 mM NaCl, 1mM EDTA, 1% Glycerol, 0.04% Tween-20, 0.1% BSA) for 120s to obtain a baseline reading, then the biosensors were dipped into wells containing the various concentrations of compounds for 100s, which was followed by a 120s buffer wash to allow the dissociation of compounds from the sensor. Global fitting of the binding curves generated a best fit with the 1:1 model and the kinetic association and dissociation constants were calculated. The systematic baseline drifts were corrected by double subtracting the shifts recorded from sensors loaded with ligands but incubated with no analytes and anther reference sensor loaded without ligands but running the same assay. All binding experiments were performed in black 96-well plates (Gerner) containing 200μl of assay buffer in each well at 25 °C with an agitation speed of 1000 rpm. Curve fitting, steady state analysis, and calculation of kinetic parameters (kon, koff and K_D_) and Rmax parameters were done using Octet software version 9.0 (ForteBio). The goodness of fit for the binding data was assessed by evaluation of the χ^2^ and R^2^values generated from all the fitting analysis. Two independent experiments were performed for each sample.

Cell-based antiviral activity assay

Vero E6 cells were maintained in DMEM supplemented with 10% FBS at 37℃ and humidified 5% CO_2_. Before infection, 10,0000 Vero E6 cells were seeded into 48-well plates in DMES (10% FBS) and incubated at 37℃ and humidified 5% CO_2_. After 12h, the medium was replaced with 200μl of DMEM (2% FBS) per well containing the compound at 20μM (for primary screen) or one concentration within six gradients (for EC_50_ determination) to incubate for 2h, then SARS-CoV-2 was added at an MOI of 0.01 and then plates were incubated at 37℃ and humidified 5% CO2. At 24 hours post-infection, the supernatants were collected and the viral RNA in supernatants was extracted and then in reverse transcription using PrimeScript RT reagent Kit with gDNA Eraser (TaKaRa). For determining the viral copies, absolute quantitative RT-PCR was performed with TB Green® Premix Ex TaqTM II (TaKaRa). The primers used for qRT-PCR were RBD-qF1: 5′-CAATGGTTTAACAGGCACAGG-3′ and RBD-qR1:5′-CTCAAGTGTCTGTGGATCACG-3’. All experiments involving SARS-CoV-2 were conducted in BSL3 facility of Wuhan Institute of Virology, Chinese Academy of Sciences. Three independent experiments of each compound determining EC_50_ values were performed, and EC_50_ values were fitted and calculated in GraphPad Prism software version 8 (GraphPad Software Inc., San Diego, CA).

For cytotoxicity measurement, Vero E6 cells were seeded to a 96-well plate (20,000 cells/well), then added with medium containing gradient concentrations of compoundsat 100 μL/well next day. The cytotoxicity was determined after 24h using the CCK8 assay kit, and CC_50_ values of nucleoside analogs were calculated with Graphpad Prism software 8.0 (GraphPad Software Inc., San Diego, CA).

Determination of Antiviral Activity of Compound JZD-07 *in Vivo*

Transgenic K18-hACE2 mice aged 7-8 weeks were purchased from Jiangsu GemPharmatech Biotechnology Co., Ltd. (Jiangsu, China). For virus challenge, mice were intranasally infected with 1 × 10^3^ PFU of SARS-CoV-2 delta variant per mouse. After 2 hours of viral challenge, mice were intraperitoneally injected with compound **JZD-07** with dose of 300mpk or vehicle (day 0). After day 0, mice were intraperitoneally administered with compound **JZD-07** twice daily (BID) at day 1 and day 2. At day 2, mice were sacrificed, and lung tissues were collected for viral copies and viral titer detection.

Fixed tissue samples were used for immunohistochemistry (IHC) for the detection of the SARS-CoV-2 antigen (SARS-CoV-2 Nucleocapsid Protein (HL344) Rabbit mAb #26369, CST). The image information was collected using a Pannoramic MIDI system (3DHISTECH, Budapest) and FV1200 confocal microscopy (Olympus).

All the K18-hACE2 mice were cared following the recommendations of National Institutes of Health Guidelines for the Care and Use of Experimental Animals. Viral infections were performed in biosafety level 3 (BSL-3) facility. (Ethics number: WIVA25202202).

Protein crystallization and structure determination

To obtain the SARS-CoV-2 3CL^pro^ with no extra residue at the N-terminus, the cDNA of full-length SARS-CoV-2 3CL^pro^ (GenBank: MN908947.3) was cloned into the pET-15b vector with an N-terminal 6 × His-SUMO2 fusion tag. The resulting plasmid was then transformed into *E. coli* strain BL21 (DE3) for protein expression. The expressed fusion protein was purified by a Ni-NTA column (GE Healthcare) and then cleaved by the SUMO specific peptidase 2 (SENP2) to remove the 6 × His-SUMO2 fusion tag. The resulting protein sample was further purified using a Q-Sepharose column (GE Healthcare) followed by a size-exclusion chromatography (HiLoad^TM^ 16/600 Superdex^TM^ 200 pg, GE Healthcare). The eluted 3CL^pro^ were stored in a solution (10 mM Tris, pH 7.5) for protein crystallization.

The purified SARS-CoV-2 3CL^pro^ protein was concentrated to 9 mg/mL for crystallization. To obtain complex structures, the SARS-CoV-2 3CL^pro^ protein was incubated with 5 mM compound **JZD-07** for 1h before crystallization condition screening. Crystallization was performed at 20 ^o^ C using a hanging drop vapor-diffusion method by mixing equal volumes (1.5:1.5 μL) of the protein mixture and reservoir solution. Crystals of the complex was obtained under the condition of 10-25% PEG6000, 100 mM MES, pH 5.75-6.25, and 3% DMSO. Crystals were cryo-protected by briefly soaking in reservoir solution supplemented with 20% (v/v) glycerol before flash-freezing in liquid nitrogen. X-ray diffraction data were collected at beamline BL19U1 at the Shanghai Synchrotron Radiation Facility^4^. Bluice was used to collect X-ray diffraction. The data were processed with HKL2000 software packages.^5^ The complex structures were solved by molecular replacement using the program PHASER^6^ with a search model of PDB code 6M2N. The model was built using Coot^7^ and refined with XYZ (reciprocal-space), individual B factors, TLS parameters, and occupancies implemented in the program PHENIX.^8^ The refined structures were deposited to Protein Data Bank with accession codes listed in Table S4. The complete statistics as well as the quality of the solved structure are also shown in Table S4. All structural figures were generated using Pymol software.

General chemistry

Unless otherwise specified, commercially available reagents were purchased from commercial sources and used without further purification. Analytical thin layer chromatography (TLC) was performed on HSGF 254 (0.15–0.2 mm thickness), visualized by irradiation with UV light (254 nm). Column chromatography was performed on silica gel FCP 200-400 or 300-400. Products were characterized by their NMR and HRMS spectra. ^1^H and ^13^C NMR spectra were recorded on a 500, or 600 MHz instrument. The chemical shifts were reported in parts per million (ppm, δ) downfield from tetramethylsilane (TMS). Proton coupling patterns were described as singlet (s), doublet (d), triplet (t), quartet (q), multiplet (m), doublet of doublets (dd), and broad (br). High-resolution mass spectra (HRMS) were measured on a Micromass Ultra Q-TOF spectrometer. See the supplementary information for the specific synthesis process of the compounds.

Synthesis for compounds JZD-07

As shown in Scheme 1, the key intermediate **4** can be prepared according to the synthetic route from the literature^9^, the intermediate then further coupled with 3,4-dichlorobromobenzene to obtain **5**, followed by amide condensation to obtain compounds **JZD-07**.

**Scheme 1**. Synthesis Procedure of **JZD-07**^a^

**^a^Reagents and conditions**: (a) HATU, morpholine, DIPEA, DMF; (b) BH_3_, reflux, THF; (c) HCl, EtOAc; (d) 4-bromo-1,2-dichlorobenzene, Pd_2_dba_3_, X-phos, Cs_2_CO_3_, toluene, 110 °C, 12 h; (e) HATU, DIPEA, DMF, rt.

**4-((4-(3,4-Dichlorophenyl)piperazin-2-yl)methyl)morpholine (5)**

Under argon, **4** (synthesized according to the reported route[^1^](#_ENREF_30)) (1 g, 5.4 mmol) and 3,4-dichlorobromobenzene (1.34 g, 5.94 mmol) were dissolved in toluene, followed by the addition of tridibenzylideneacetone Dipalladium (Pd_2_dba_3_, 203.0 mg, 0.22 mmol), 2-dicyclohexylphosphorus-2,4,6-triisopropylbiphenyl (X-Phos, 238.1 mg, 0.5 mmol) and cesium carbonate (3.26 g, 10 mmol), stirred at 110 °C overnight, cooled to room temperature after the reaction, and filtered, and the filter cake was washed with ethyl acetate. The combined organic phases were washed successively with saturated sodium chloride solution, dried over anhydrous sodium sulfate and concentrated to obtain a yellow oil. The oil was separated by column chromatography with dichloromethane/methanol=20:1 as an eluent to obtain a yellow oil as compound **5** (1.07 g, 60%). **^1^H NMR (600 MHz, MeOD)** *δ* 7.30 (d, *J* = 8.9 Hz, 1H), 7.05 (d, *J* = 2.9 Hz, 1H), 6.87 (dd, *J* = 9.0, 2.9 Hz, 1H), 3.70 (t, *J* = 4.6 Hz, 4H), 3.57 (dd, *J* = 21.8, 11.8 Hz, 2H), 3.09 (dt, *J* = 11.9, 2.8 Hz, 1H), 3.04 – 2.97 (m, 1H), 2.91 (td, *J* = 11.7, 3.2 Hz, 1H), 2.75 (td, *J* = 11.6, 3.1 Hz, 1H), 2.53 (d, *J* = 9.9 Hz, 2H), 2.47 – 2.34 (m, 5H). **^13^C NMR (151 MHz, MeOD)** *δ* 151.1, 132.2, 130.2, 121.5, 116.9, 115.5, 66.6, 61.1, 54.0, 52.40, 51.23, 48.4, 44.5;

**4-(4-(3,4-Dichlorophenyl)-2-(morpholinomethyl)piperazine-1-carbonyl)quinolin-2(1H)-one** (**JZD-07**)

Dissolve **5** (1.07 g, 3.24 mmol) in DMF, add 2-hydroxyquinoline-4-carboxylic acid (674 mg, 3.56 mmol), HATU (1.35 g, 3.56 mmol), DIPEA (838 mg, 6.48 mmol), stirred at room temperature for 1 h, excess water was added after the reaction, extracted three times with ethyl acetate, the organic phases were combined, washed with saturated sodium chloride solution, dried over anhydrous sodium sulfate and concentrated to obtain a yellow oil. The oil was separated by column chromatography with dichloromethane/methanol=20:1 as an eluent to obtain a yellow solid as compound **JZD-07** (812 mg, 50%).**^1^H NMR (500 MHz, CD_2_Cl_2_)** *δ* 11.79 (s, 1H), 8.11 (d, *J* = 8.0 Hz, 1H), 7.60 (dd, *J* = 13.1, 6.2 Hz, 1H), 7.42 (d, *J* = 8.2 Hz, 1H), 7.34 – 7.25 (m, 2H), 7.00 (d, *J* = 2.7 Hz, 1H), 6.77 (dd, *J* = 8.9, 2.8 Hz, 1H), 6.74 – 6.60 (m, 1H), 3.89 – 3.62 (m, 5H), 3.50 – 3.33 (m, 3H), 3.15 – 2.97 (m, 2H), 2.93 – 2.45 (m, 6H), 2.32 (s, 1H). **^13^C NMR (126 MHz, CD_2_Cl_2_)** *δ* 165.3, 162.8, 150.3, 147.6, 138.5, 138.3, 132.2, 131.1, 130.1, 125.9, 122.7, 122.3, 118.0, 117.6, 115.8, 115.6, 66.5, 56.9, 53.9, 50.34, 48.3, 45.3, 41.8. **HRMS-ESI m/z** [M + H]^+^ calculated for C_25_H_27_Cl_2_N_4_O_3_: 501.1455, found: 501.1461. Melting point 224 ℃. purity, 97.1% (HPLC).

**HPLC spectrum**

solvent A: CH_3_OH; solvent B: water; A/B = 70/30 (v/v), flow rate: 1.0 mL/min, at 254 nm.


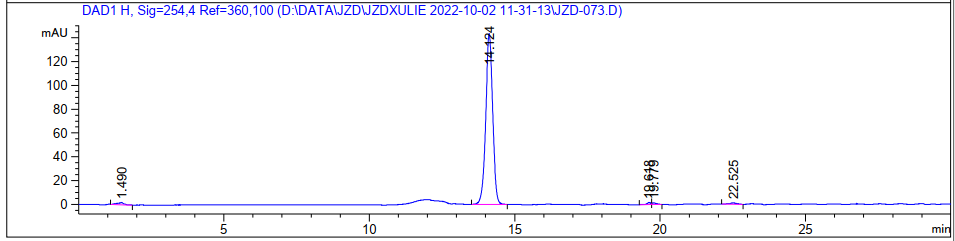


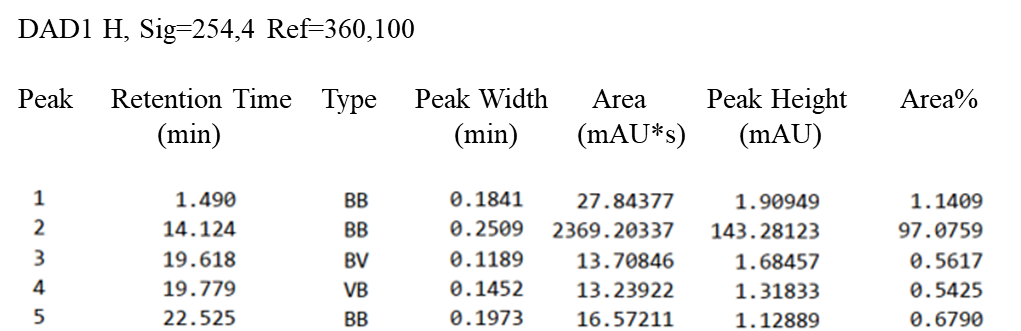


**Supplementary Figures**


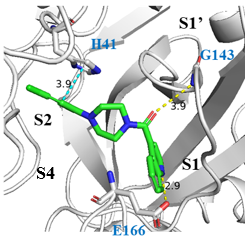


Supplementary Fig. 1 G006 docking mode analysis. Hydrogen bonds are indicated as yellow dashed lines; π−π interaction is indicated as a cyan dashed line (PDB ID:6LU7).

a


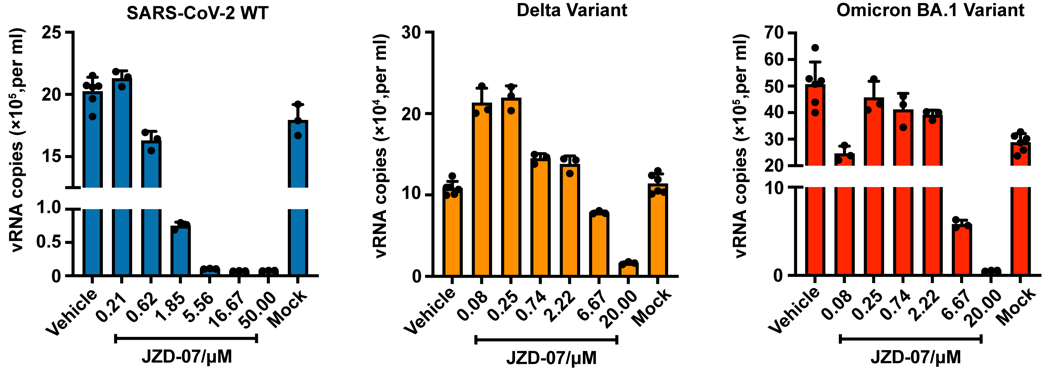


**b**

**
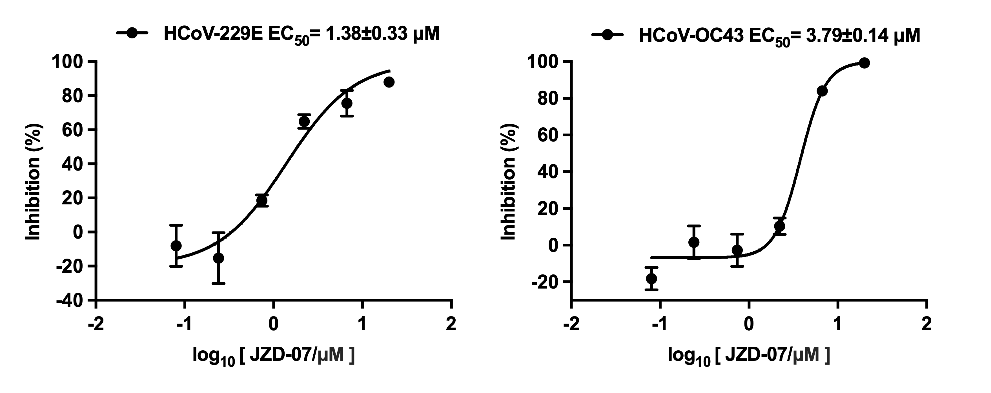
**

Supplementary Fig. 2 (a) In vitro antiviral activities of Compound JZD-07 against SARS-CoV-2 and Delta/Omicron variants. Inhibition of original strain (blue), Delta variant (yellow) and Omicron variant (red) replication by Compound JZD-07 in Vero E6 cells. Dose–response curves for Compound JZD-07 in Cell-based antiviral activity assay (EC_50_) The Y-axis of the graphs represent viral RNA copies (per mL). The quantification of absolute viral RNA copies (per mL) in the supernatant at 24 h post infection determined by qRT-PCR analysis. Data are shown as mean ± SD, n = 3 biological replicates. (b) In vitro antiviral activities of Compound JZD-07 against other human coronaviruses. Data are shown as mean ± SD, n = 3 biological replicates.

Supplementary Fig. 3 Characterization of the mechanism of action of JZD-07, ML-188^10^ and CCF-981^11^ to SARS-CoV-2 3CL^pro^ using enzyme kinetic studies. Kinetic parameters in the presence of various concentrations of JZD-07, ML-188 and CCF-981 were globally fitted with a Michaelis−Menten function in prism 8.


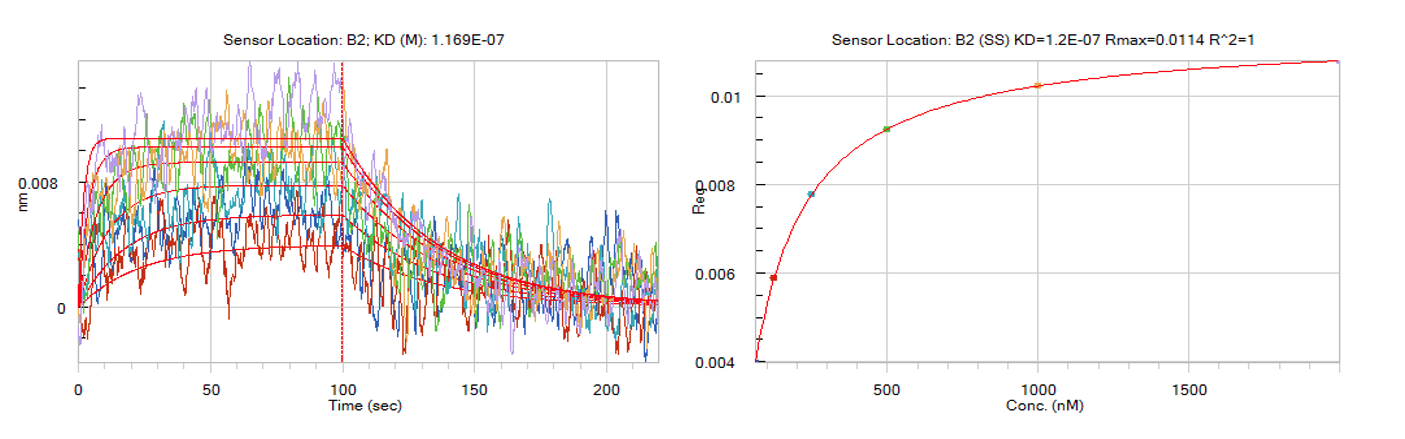


Supplementary Fig. 4 BLI analyses of the binding of Compound JZD-07 to SARS-CoV-2 3CL^pro^. Steady-state analysis was performed using the average signal measured at the end of the association step (between 90 and 100 s) and *K*_D_ value was determined with equation: R_eq_ = R_max_ * [B] / (K_D_+[B]), fitting the curves with R_eq_ of each concentration.

**
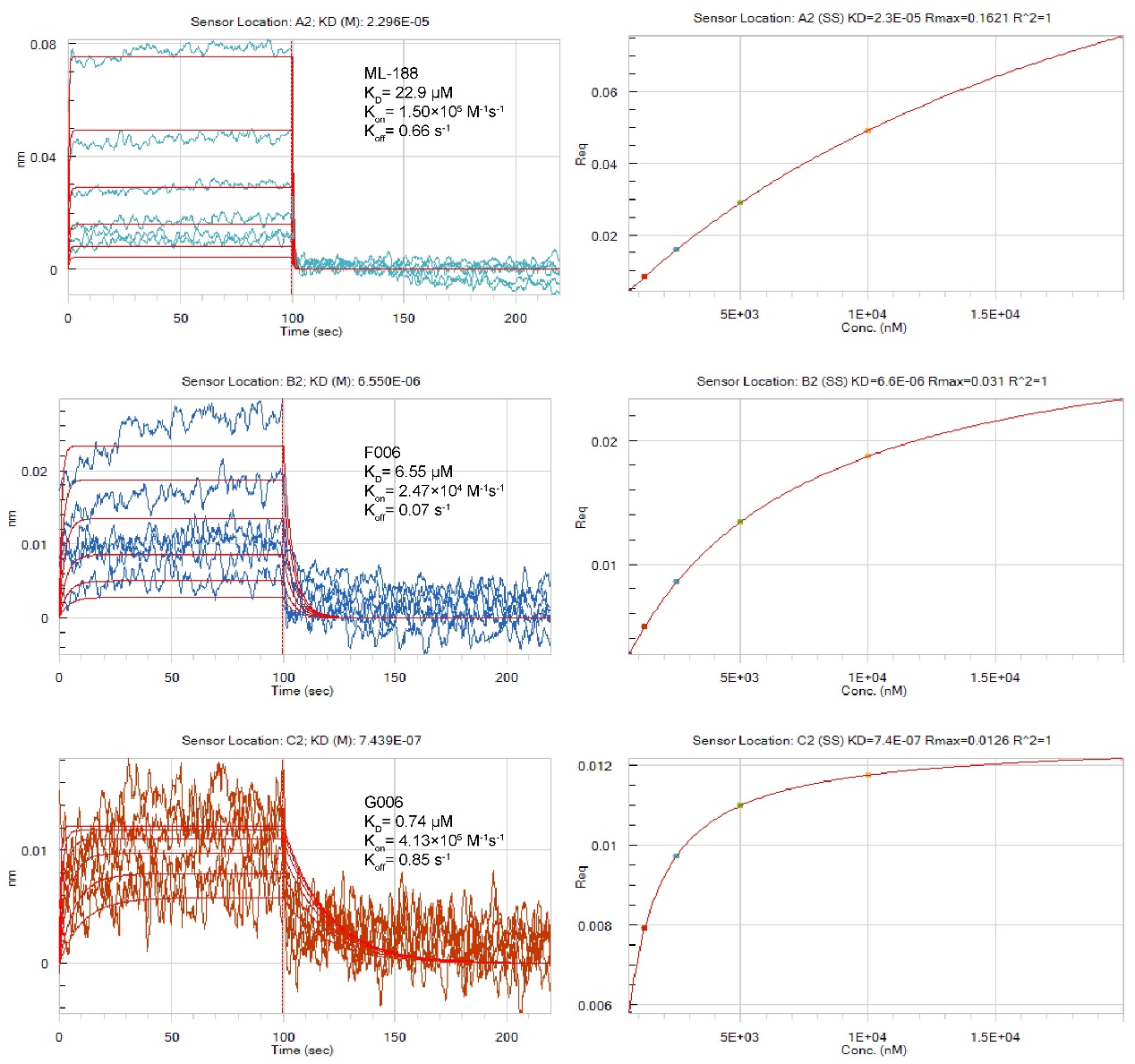

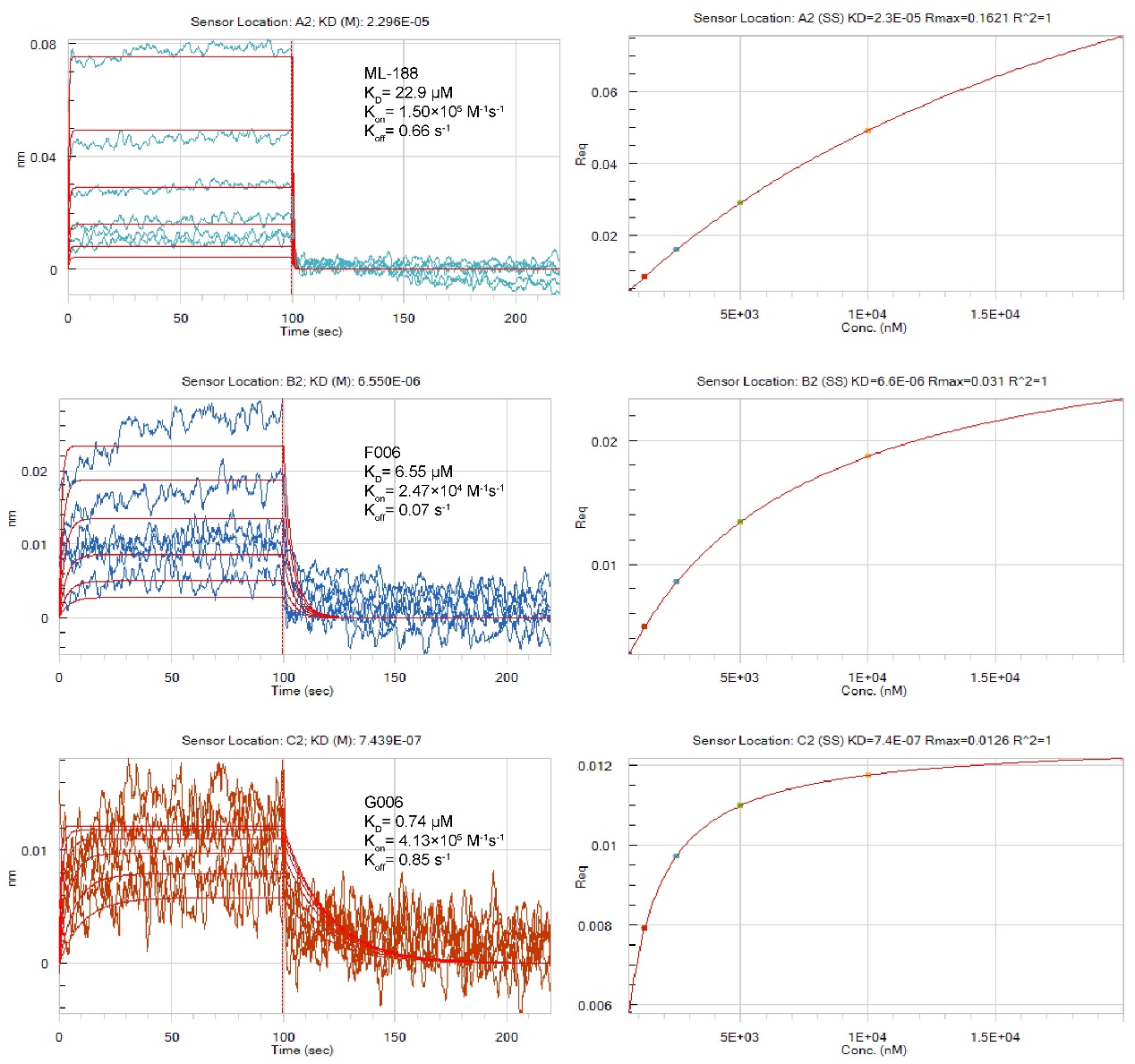
**

Supplementary Fig. 5 BLI analyses of the binding of ML-188/ G006 to SARS-CoV-2 3CL^pro^. The *K*_D_ value was determined by Steady State Analysis using Fortebio Data Analysis 9.0.


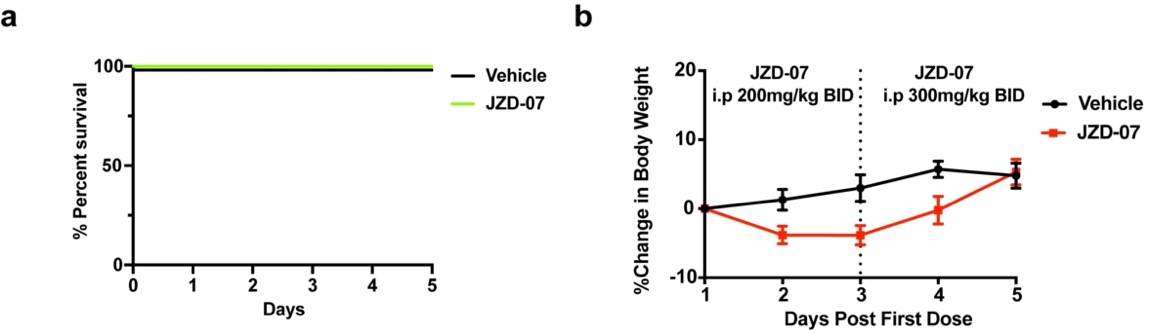


Supplementary Fig. 6 Survival curve (a) and mouse body weight change (b) in mice when treated with either vehicle or JZD-07 at 200mg/kg twice per day (BID) intraperitoneally (i.p.) first 3 days and at 300mg/kg twice per day (BID) intraperitoneally (i.p.) last 2 days. No mice died or significant body weight lost (>10%).

**Supplementary Table 1.** Selectivity data of SARS-CoV-2 3CL^pro^ inhibitors

| Portein Name | **GC-376** | **PF-07321332** | **JZD-07** |
| --- | --- | --- | --- |
|  | **IC_50_ (μmol)** | | |
| SARS-CoV-2 PL^pro^ | N.D. | N.D. | >100 |
| SARS-CoV-2 RdRp | N.D. | N.D. | >100 |
| SARS-CoV-2 nsp16/10 | N.D. | N.D. | >100 |
| Calpain 1/cysteine | 0.02 | >100 | >100 |
| Cathepsin B/cysteine | 0.09 | >100 | >100 |
| Cathepsin L/cysteine | 0.002 | 8.36 | >100 |
| Cathepsin K/cysteine | 0.0004 | 0.585 | >100 |
| Cathepsin D/aspartyl | >100 | >100 | >100 |
| Proteasome | - | - | >100 |
| Caspase3/ cysteine | >100 | >100 | >100 |
| Thrombin/serine | >100 | >100 | >100 |
| Trypsin/serine | >100 | >100 | >100 |
| DPP-4/cysteine | >100 | >100 | >100 |

^a^Positive controls used for the corresponding enzymes are listed below: SARS-CoV-2 PL^pro^: GRL0617 IC_50_= 1.03±0.22 μM; SARS-CoV-2 RdRp: suramin IC_50_= 364.5±3.0 nM; SARS-CoV-2 nsp16/10: 2a^12^ IC_50_= 17.1 nM; Cathepsin B: E-64 IC_50_= 13.4±1.4 nM; Cathepsin K: E-64 IC_50_= 13.6±5.0 nM; Cathepsin L: E-64 IC_50_= 65.0±8.9 nM; Proteasome: PS-341 IC_50_= 14.7±3.4 nM Caspase3: Ac-DEVD-CHO IC_50_= 1.7±0.3 nM; Thrombin: Argatroban IC_50_= 6.0±1.3 nM; DPP-4: MK0431 IC_50_= 12.3±1.0 nM; Trypsin: Camostat mesylate IC_50_= 101.0±26.4 nM.

**Supplementary Table 2.** Crystallography data collection and refinement statistics

|  | | 3CL^pro^- **JZD-07** |
| --- | --- | --- |
| **PDB ID** | | 8GTV |
| Space Group | | P 1 21 1 |
| Cell Dimension: a (Å) | | 51.65 |
| b (Å) | | 82.13 |
| c (Å) | | 89.12 |
| Wavelength (Å) | | 0.9785 |
| Reflections (unique) | | 68497 |
| Resolution Range (Å) | | 50.00-1.80 |
| Highest-Resolution Shell (Å) | | 1.86-1.80 |
| Redundancy | | 6.5 (5.5) |
| I/σ (I) | | 21.4 (1.3) |
| Completeness (%) | | 99.8 (99.1) |
| Rwork/Rfree | | 0.2239/0.2469 |
| Clashscore | | 1.71 |
| MolProbity Score | | 0.93 |
| **RMS** V**alues** | | |
| Bond length (Å) | | 0.007 |
| Bond angle (°) | | 0.892 |
| **Numbers of Non-hydrogen Atoms** | | |
| Protein | | 4452 |
| Inhibitor | | 68 |
| Water Oxygen | | 155 |
| Others | | 0 |
| **B-factor (Å^2^)** |  |  |
| Protein | | 44.26 |
| Inhibitor | | 43.42 |
| Water Oxygen | | 41.01 |
| **Ramachandran plot** | | |
| Favored (%) | | 98.49 |
| Allowed (%) | | 1.51 |
| Outliers (%) | | 0 |

Reference

1. Li, D. et al. Development of Macrocyclic Peptides Containing Epoxyketone with Oral Availability as Proteasome Inhibitors. *J Med Chem* **61**, 9177-9204 (2018).

2. Li, S. et al. Discovery and Rational Design of Natural-Product-Derived 2-Phenyl-3,4-dihydro-2H-benzo[f]chromen-3-amine Analogs as Novel and Potent Dipeptidyl Peptidase 4 (DPP-4) Inhibitors for the Treatment of Type 2 Diabetes. *J Med Chem* **59**, 6772-6790 (2016).

3. Du, J. Q. et al. Isoquinoline-1,3,4-trione derivatives inactivate caspase-3 by generation of reactive oxygen species. *J Biol Chem* **283**, 30205-15 (2008).

4. Zhang, W.-Z. et al. The protein complex crystallography beamline (BL19U1) at the Shanghai Synchrotron Radiation Facility. *Nucl Sci Tech* **30**, 170 (2019).

5. Zbyszek Otwinowski et al. Processing of X-ray diffraction data collected in oscillation mode. *Methods In Enzymology* **276**, 307-326 (1997).

6. McCoy, A. J. et al. Phaser crystallographic software. *J Appl Crystallogr* **40**, 658-674 (2007).

7. Emsley, P. et al. Coot: model-building tools for molecular graphics. *Acta Crystallogr D Biol Crystallogr* **60**, 2126-32 (2004).

8. Adams, P. D. et al. PHENIX: building new software for automated crystallographic structure determination. *Acta Crystallogr D Biol Crystallogr* **58**, 1948-54 (2002).

9. Ashton, K. S. et al. Small molecule disruptors of the glucokinase-glucokinase regulatory protein interaction: 1. Discovery of a novel tool compound for in vivo proof-of-concept. *J Med Chem* **57**, 309-24 (2014).

10. Kitamura, N. et al. Expedited Approach toward the Rational Design of Noncovalent SARS-CoV-2 Main Protease Inhibitors. *J Med Chem* **65**, 2848-2865 (2022).

11. Han, S. H. et al. Structure-Based Optimization of ML300-Derived, Noncovalent Inhibitors Targeting the Severe Acute Respiratory Syndrome Coronavirus 3CL Protease (SARS-CoV-2 3CL(pro)). *J Med Chem* **65**, 2880-2904 (2022).

12. Bobileva, O. et al. Potent SARS-CoV-2 mRNA Cap Methyltransferase Inhibitors by Bioisosteric Replacement of Methionine in SAM Cosubstrate. *ACS Med Chem Lett* **12**, 1102-1107 (2021).
